# Study of Correlation of Pre-Operative Findings with Intra-Operative Ossicular Status in Patients with Chronic Otitis Media

**Published:** 2018-09

**Authors:** Pragya Singh, Shraddha Jain, Disha Methwani, Sanika Kalambe, Deepshikha Chandravanshi, Sagar Gaurkar, Prasad T Deshmukh

**Affiliations:** 1 *Department of Otorhinolaryngology and Head and Neck Surgery, Jawahar Lal Nehru Medical College, Datta Meghe Institute of Medical Sciences (DU), Sawangi, Wardha, Maharashtra, India.*

**Keywords:** Chronic otitis media, Granulations, Mucosal otitis media, Necrosis, Ossicles, Pars flaccida cholesteatoma, Pars tensa cholesteatoma, Squamous otitis media, Tympanomastoidectomy

## Abstract

**Introduction:**

Chronic otitis media (COM) has been broadly classified into mucosal and squamous subtypes. COM types are associated with erosion of the ossicular chain. The aim of the present study was to correlate the type of COM, the site of perforation/retraction, and the type of disease pathology with the pattern and degree of ossicular chain necrosis.

**Materials and Methods:**

A prospective cross-sectional study was performed in 76 cases of COM, who were subjected to tympanomastoidectomy. Pre-operative findings were compared with per-operative ossicular chain status and pathology.

**Results:**

Incus was found to be the most vulnerable ossicle for erosion, followed by malleus and suprastructure of stapes. The pattern of multiple ossicle involvement was more common. Ossicular chain erosion was more common in squamous COM than mucosal COM (X^2^=66.25; P=0.0001) and in the presence of cholesteatoma and granulations. Ossicular necrosis was most common in squamous disease with cholesteatoma, followed by squamous disease with granulations, mucosal disease with granulations, and inactive mucosal disease in that order.

**Conclusion:**

The degree of ossicular necrosis has a positive correlation with the type of disease pathology, being higher in squamous disease than in mucosal disease. The pattern of ossicular necrosis varies with the site of origin of the disease and the pattern of spread of cholesteatoma, being variable for pars tensa and pars flaccida squamous disease.

## Introduction

Chronic otitis media (COM) is a permanent abnormality of the pars tensa (PT) or pars flaccida (PF), which may manifest in the form of atelectasis, dimer formation, perforation, tympanosclerosis, retraction pocket development or cholesteatoma (1). COM is most often a result of earlier acute otitis media, negative middle air pressure, or otitis media with effusion and has almost replaced the classic term “chronic suppurative otitis media” (CSOM), which is no longer advocated as COM is not necessarily a result of the gathering of pus (2). The Browning classification of COM broadly divides COM into mucosal and squamous subtypes, depending upon whether it is a perforation in the PT in the former or a retraction pocket/frank cholesteatoma in the latter type (2). It has been further subdivided into active and inactive forms. This classification is now more accepted than the former one comprising tubotympanic or safe CSOM and atticoantral or unsafe CSOM, which were defined as “intermittent or persistent, chronic purulent drainage through a perforated tympanic membrane, which can be associated with cholesteatoma” (3).

COM of all types is associated with erosion of the ossicular chain (4). The incidence and degree of ossicular destruction is much greater in cases of unsafe CSOM, due to the presence of cholesteatoma and/or granulations (5). Although bone erosion may occur in COM without cholesteatoma, it is more frequent when the keratinizing epithelium is present (6). Various factors that have been implicated in bone erosion in human cholesteatoma including osteoclasts, pressure necrosis, collagenolytic enzyme, tumor necrosis factor (TNF)-α, lysosomal enzymes, and non-lysosomal enzymes calpain I and II, which contribute to collagen destruction (7–10). Ossicular necrosis has been observed in all forms of COM.

Knowledge of the factors contributing to ossicular necrosis and the pattern of ossicular involvement is helpful in planning reconstructive surgery. To the best of our knowledge, no studies have been performed which have correlated the pattern of ossicular necrosis with different types of COM, *viz*. squamous and mucosal types and the site and type of disease pathology in detail. Hence, the present study was undertaken to correlate different types of COM, the site of perforation/retraction, and the type of disease pathology with the per-operative pattern and degree of ossicular chain necrosis.

## Materials and Methods

A prospective cross-sectional study was carried out from 2014 to 2016. Initially, a total of 86 COM patients attending the ear, nose, and throat (ENT) outpatients, inpatients and casualty departments of Acharya Vinoba Bhave Rural Hospital, Sawangi (Meghe), Wardha, were shortlisted for our study. After applying inclusion criteria (COM patients aged 1–70 years) and exclusion criteria (patients with previous history of ear surgery, known immunodeficiency disorder, Down’s syndrome, craniofacial anomalies, trauma to ear, complete or total hearing loss and tuberculous otitis media), 76 patients were found to be suitable and were selected for the present study. Written informed consent and prior approval of the Institutional ethics committee [Approval letter no. DMIMS (DU)/IEC/2014-15/814] was obtained. 

**Fig 1:**
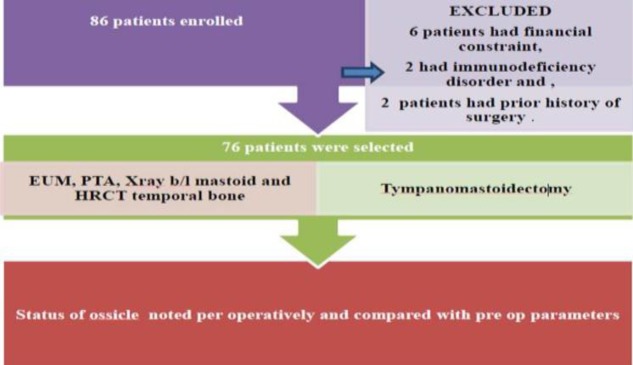
Flow chart

In all included patients, a detailed history was obtained, which was followed by a general systemic examination and a detailed ENT evaluation with the help of a Bull’s lamp, a head mirror, otoscope and microscope. With special emphasis on examination of the middle ear by otoscopy and otomicroscopy (Karl Kaps D 35614 Asslar Europastrasse), documentation regarding the site and type of retraction and perforation was carefully performed, and classification of type of COM was performed according to the Browning classification. The status of the middle ear mucosa and the presence of any adhesions, myringosclerosis and granulations, if any, were assessed. Apart from basic investigations, cases selected for our study were subjected to pure tone audiometry and radiological investigations, as indicated.

**Fig 2:**
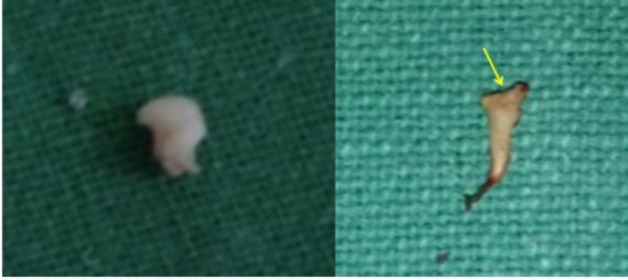
Remnant part of head of malleus on the left and absent head of malleus on the right (yellow arrow).

**Fig 3:**
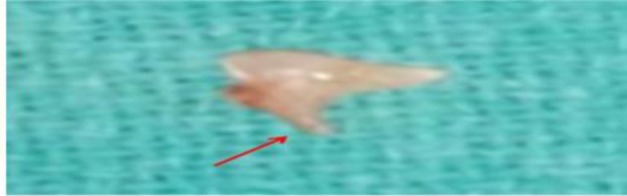
Erosion of lenticular process of incus (red arrow)

**Fig 4:**
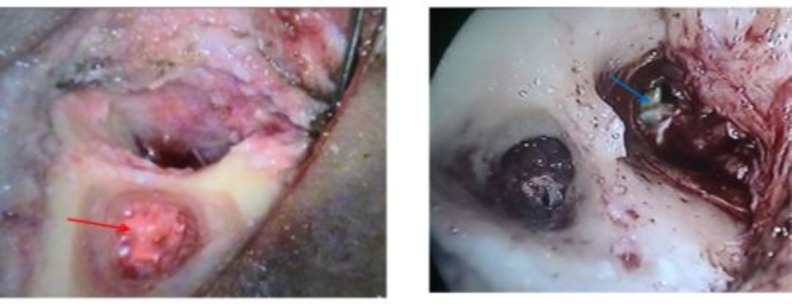
Per-operative findings during tympanomasto- idectomy showing presence of granulation disease (red arrow) and cholesteatoma sac (blue arrow)

All patients underwent tympanoplasty, with or without canal wall up or canal wall down mastoidectomy, depending upon the type and extent of disease. For each patient, the status of each ossicle and the disease pathology were evaluated and described intraoperatively (Figs.2,3,4). Findings thus obtained were entered in the proforma for the study. Disease pathology was confirmed by histopathological examination. A Chi-square test was used to evaluate the level of significance and P<0.05 was considered statistically significant.

## Results

Of 76 COM patients, 53.9% were females, with the highest proportion of patients being in the 16–30-year age group (40.79%). All patients had a positive history of otorrhea; 36 (47.4%) patients had mucosal COM and 40 (52.6%) patients had squamous COM. All other baseline characteristics are shown in (Table.1).

**Table 1:** Baseline characteristics

**Characteristics**		**Number (%) of patients**
Age in years		
1–15		8 (10.53%)
6–30		31 (40.79%)
31–45		21 (27.63%)
46–60		13 (17.11%)
61–75		3 (3.95%)
Mean ± SD (range)		32.40±14.94 (8–70 years)
Sex		
Male		35 (46.1%)
Female		41 (53.9%)
Laterality of disease		
Right		17 (22.4%)
Left		34 (44.7%)
Bilateral		25 (32.9%)
Laterality of ear operated		
Right		26 (34.2%)
Left		50 (65.8%)
Symptoms		
Otorrhea		76 (100.00%)
Hearing loss		68 (89.47%)
Otalgia		9 (11.84%)
Tinnitus		23 (30.26%)
Vertigo		1 (1.32%)
Facial weakness		0 (0.00%)
Swelling behind ear		3 (3.95%)
Fever with chills & rigors		0 (0.00%)
Type of disease		
Mucosal		36 (47.4%)
Squamous		40 (52.6%)
Otoscopic findings		
Active Mucosal		10 (13.2%)
Inactive Mucosal		26 (34.2%)
Pars Tensa Squamous	Active	26 (34.2%)
	Inactive	0 (0.00%)
Pars Flaccida Squamous	Active	14 (18.4%)
	Inactive	0 (0.00%)

Per operatively, mucosal COM was further classified as “mucosal COM without granulation” (34.2% patients) or “mucosal COM with granulation” (13.2% patients). Patients with PT squamous COM were further classified into two groups, “with exclusive granulation disease” in 22.4% patients and “with cholesteatoma” in 11.8% patients. Patients with PF disease were also grouped into PF cholesteatoma and PF granulation disease, the distribution of which is shown in (Table.2).

**Table 2:** Per-operative pathology

**Per-operative findings**	**Number (%) of patients**
Mucosal without granulation	26 (34.2%)
Mucosal with granulation	10 (13.2%)
Pars tensa squamous with granulation only	17 (22.45%)
Pars tensa squamous with cholesteatoma and with/without granulation	9 (11.8%)
Pars flaccida squamous with granulation only	0 (0.00%)
Pars flaccida squamous with cholesteatoma and with/without granulation	14 (18.4%)

Overall, 48.68% patients had an intact ossicular chain and in 51.32% patients, one or more than one ossicle was found to be necrosed. Incus was found to be the most vulnerable ossicle for erosion in 47.37% patients, followed by malleus, showing necrosis in 38.16% followed by stapes suprastructure necrosis in 35.53% patients. Multiple ossicle involvement was found in 26.32% patients and isolated incus necrosis was seen in only 5.26% patients. The pattern of ossicular necrosis of the rest of the ossicles is depicted in Table 3.

**Table 3:** Ossicular necrosis

**Ossicles**		**Number (%) of patients**		
All intact		37 (48.68%)		
One or more necrosed		39 (51.32%)		
**Ossicles**	**Malleus**		**Incus**	**Stapes**
Intact	47 (61.84%)		40 (52.63%)	49 (64.47%)
Necrosed	29 (38.16%)		36 (47.37%)	27 (35.53%)
Total	76 (100%)		76 (100%)	76 (100%)
**Erosion of Ossicles**	**Number (%) of patients**			
Incus	4 (5.26%)			
Malleus + incus	6 (7.89%)			
Malleus + stapes	9 (11.84%)			
Malleus + incus + stapes	20 (26.32%)			
No ossicle involvement	37 (48.68%)			

The overall incidence of ossicular necrosis was found to be highest in active squamous disease with cholesteatoma, followed by PT squamous disease with granulation, mucosal disease with granulation, and absent in inactive mucosal COM. In patients with inactive mucosal COM, there was no ossicular necrosis, whereas in active mucosal COM with granulation, ossicular necrosis was found in 30% of patients. In 40 patients with squamous COM, 90% had ossicular chain erosion, whereas among 36 patients of mucosal chronic otitis media, the majority of patients had an intact and mobile ossicular chain (91.67%), with only 8.33% patients with ossicular chain necrosis, in the presence of granulation disease (X^2^=66.25; P=0.0001) (Table.4).

**Table 4:** Correlation of the type of disease with ossicular chain status

**Type of disease**	**Ossicular necrosis**		**Total**
	Present	Absent	
Mucosal	3 (8.33%)	33 (91.67%)	36
Squamous	36 (90%)	4 (10%)	40
X^2^-value	66.25, P=0.0001		

In patients with PT squamous disease, 15.38% patients had an intact ossicular chain and 84.62% patients had ossicular chain necrosis. In contrast, all 14 (100%) patients of PF squamous COM had ossicular chain necrosis, and none were found to have an intact ossicular chain (X^2^=0.989; P=0.32) (Table.5).

**Table 5:** Correlation of pars tensa and pars flaccida disease with ossicular chain status

**Type of disease**	**Ossicular necrosis**		**Total**
	**Present**	**Absent**	
Pars tensa squamous	22 (84.62%)	4 (15.38%)	26
Pars flaccida squamous	14 (100%)	0 (0%)	14
X^2^-value	0.989, P=0.3200		

**Fig 5:**
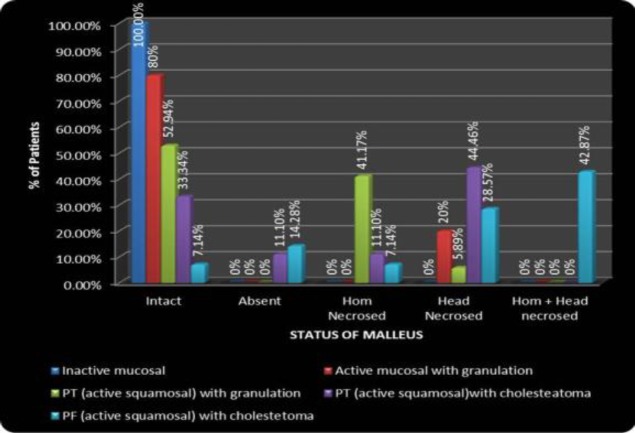
Correlation of type of disease with per-operative status of malleus

Figure 5 shows the per-operative status of malleus in different types of COM. Necrosis of malleus was most commonly seen in squamous disease.

 In our study, malleus was found to be intact in all cases of inactive mucosal COM (26 patients [100%]). Out of 10 patients with active mucosal disease with granulation, eight (80%) had an intact malleus and two (20%) had necrosis over the head of malleus. In 17 patients with PT active squamous COM with granulation, nine (52.94%) had an intact malleus, seven (41.17%) had necrosed handle of malleus and only one (5.89%) patient showed necrosis over the head of malleus. In PT active squamous disease with cholesteatoma, malleus head was found to be intact in 44.53% patients. Handle of malleus was preserved in PT squamous disease with cholesteatoma in 77.78% of patients. Only 7.14% patients with PF active squamous disease were found to have an intact malleus. In cases of PF active squamous disease, with cholesteatoma, except for two patients out of 14 (14.28%), all had absent head of malleus.

**Fig 6:**
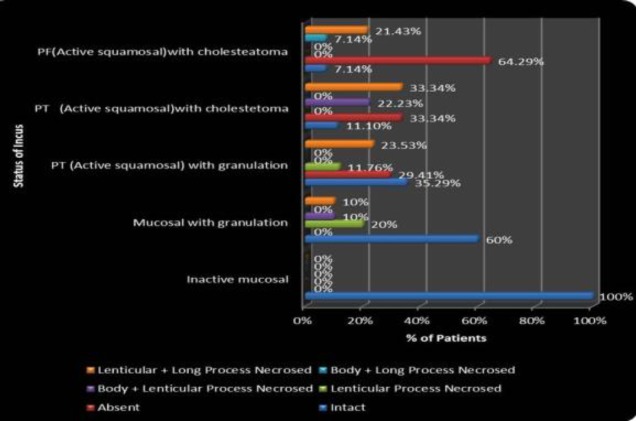
Correlation of type of disease with per-operative status of incus

**Fig 7:**
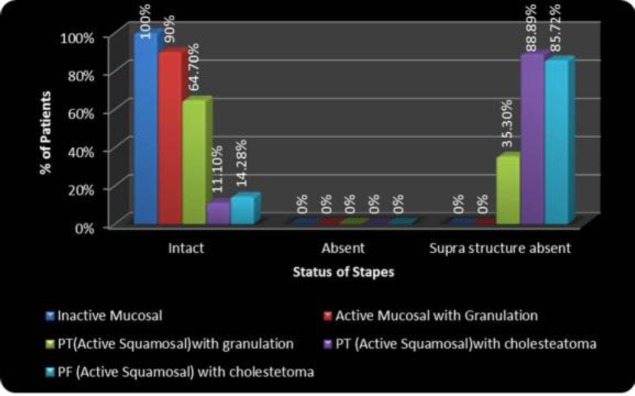
Correlation of type of disease with per-operative status of stapes

The per-operative status of incus and stapes is depicted in Figures 6 and 7, respectively. Incus was found to be intact in all cases of inactive mucosal (26 [100%] patients). 

Out of 10 patients with active mucosal disease with granulation six (60%) had an intact incus, two (20%) had necrosis over the lenticular process, one (10%) had necrosis of the body along with the lenticular process and one (10%) patient had necrosis of the long process along with the lenticular process. In 17 patients of PT active squamous COM with granulation, six (35.29%) patients had an intact incus, in five (29.41%) patients the incus was absent, two (11.76%) patients had necrosis over the lenticular process, and four (23.53%) patients had necrosis of the long process along with the lenticular process. In 64.7% patients of PT active squamous disease with granulation, stapes was found to be intact. PT active squamous disease with cholesteatoma and PF active squamous disease with cholesteatoma had almost a similar degree of incus and stapes necrosis. In patients with PF active squamous disease with cholesteatoma, incus was found to be necrosed in 92.85% cases, whereas in patients with PT active squamous disease with cholesteatoma, it was necrosed in 88.89% cases. In PF cholesteatoma cases, incus was absent in 64.29% patients and in PT cholesteatoma cases, it was absent in 33.34% cases. Stapes suprastructure was found to be necrosed in 88.89% cases in PT cholesteatoma, whereas in PF cholesteatoma, it was found necrosed in 85.72% cases.

## Discussion

In the present study, we observed a very wide age range. The greatest proportion of patients in our study were in the age group of 16–30 years. This was similar to study by Varshney et al. (11). In our study, the mean age of 32.40 years was slightly higher than in other studies, and this could be attributable to late presentation due to a lack of awareness or financial constraints as ours is a rural tertiary center.

In our study, the number of female patients slightly outnumbered the male patients. This was similar to studies by Gomaa et al. and Ginni et al. (12,13). However, other studies found a strong male predilection in squamous COM (males 64.7%; females 35.3%) (14,15).

No patients with inactive squamous disease were operated on during our study, as associated sino-nasal pathology was addressed first. This has been discussed in our previous study, where a mechanical type of Eustachian dysfunction due to sinonasal pathology is diagnosed in many cases of squamous otitis media (16).

In our study, all the 10 (13.2%) patients with active mucosal otitis media, who underwent tympanomastoidectomy, were found to have granulation disease per operatively. Patients with PT active squamous COM with retraction pockets, mostly in the posterosuperior quadrant, were found to have two types of pathology, either exclusive granulation disease or cholesteatoma associated with granulation disease. They were therefore classified into two groups; the first with exclusive granulation disease and the other group with cholesteatoma. As many as 17 (22.4%) cases of active squamous disease involving PT were found to have exclusive granulation disease.Patients with PF squamous disease were also grouped into PF cholesteatoma and PF granulation disease. No patients were found to have exclusive PF granulation disease during our study. 

All patients with PF squamous disease had cholesteatoma with/without granulations. Deep retraction pockets, according to the Browning classification, fall in the category of active squamous COM, which is believed to be associated with cholesteatoma.

Our study is probably the first to have correlated per-operative ossicular status with different types and sites of middle ear pathology in COM in such detail. Very few studies have correlated the per-operative ossicular status with type of disease pathology, *viz*. safe and unsafe COM (11,17 ). Studies correlating active and inactive mucosal disease have not been published, and only a few studies could be identified which have studied ossicular necrosis in different sites of pathology *viz*. PT and PF cholesteatoma (18).

*Overall ossicular necrosis in COM*

In our study, 37 (48.68%) patients had an intact ossicular chain and in 39 (51.32%) patients, one or more than one ossicle was found to be necrosed. We found few study designs similar to our study which have studied ossicular necrosis in both safe and unsafe CSOM (11,17,19). Varshney et al. found the ossicular chain to be intact in 92 (61.34%) cases, while Haidar et al. reported intact ossicular chain in 213 (76.4%) out of the 279 patients (11,17). Udaipurwala et al. found intact ossicular chain in 69 out of 145 patients (47.6%) and ossicular necrosis in 76 out of 145 patients of COM (52.4%) (19).

*Ossicular necrosis in mucosal (safe) versus squamous (unsafe) COM*

In this study, in patients with mucosal COM, only 8.33% patients had ossicular chain necrosis, whereas in squamous COM, the majority of patients 90%, had ossicular chain erosion (X^2^=66.25; P=0.0001). Our findings are similar to those of Varshney et al., who compared ossicular necrosis in safe and unsafe CSOM. In safe CSOM, 7.77% had ossicular damage, as against 85% in unsafe CSOM in their study (11). In two studies on unsafe CSOM, Dasgupta et al. also reported similar results (18,20). Haider et al. also found ossicular erosion to be more frequent in ears with cholesteatoma (69.3%) than in safe ears (13.9%) (17). Toran et al. presented results of ossicular chain necrosis in malleus and stapes, which were similar to the present study (21).

*Ossicular necrosis in pars tensa versus pars flaccida squamous COM*

In our study, in PT active squamous disease, 84.62% patients had ossicular chain necrosis, whereas all patients with PF squamous COM had ossicular chain necrosis, and none were found to have an intact ossicular chain. We were not able to identify any study which has studied ossicular necrosis after sub-classifying squamous COM based on site and type of disease pathology for comparison.

*Ossicular necrosis in active versus inactive mucosal COM*

Studies correlating ossicular chain status in active and inactive mucosal disease were not identified. In our study, the presence of granulation tissue was associated with ossicular necrosis in 80% of cases of active mucosal COM with granulation. This was found to be statistically signiﬁcant. Existing evidence has also demonstrated that middle ear granulations are signiﬁcantly associated with ossicular discontinuity (22). Our findings were in concordance with other studies (23).

*Multiplicity of ossicular necrosis*

In our study, 37 (48.68%) patients had all ossicles intact. In ossicular necrosis, the pattern of multiple ossicle involvement was common in 20 (26.32%) patients, and isolated incus necrosis was seen in four (5.26%) patients. Malleus plus incus necrosis was seen in six (7.89%) patients and nine (11.84%) patients showed necrosis of malleus with stapes.

In our study, the overall incidence of ossicular necrosis was found to be highest in active squamous disease with cholesteatoma, followed by PT squamous disease with granulation, and mucosal disease with granulation, and was absent in inactive mucosal COM.

*Pattern of necrosis of malleus in different types of COM*

Necrosis of malleus was most commonly seen in squamous disease. Only 7.14% patients of PF active squamous disease were found to have an intact malleus. In PT active squamous disease with cholesteatoma, malleus head was found to be intact in 44.53% patients. In patients with PT active squamous disease with granulation, the head of malleus was found to be necrosed in only 5.89% cases, whereas the necrosis of handle of malleus was seen in 41.17% cases. The spread of cholesteatoma from PT into attic is greater in the posterior direction toward aditus; therefore, the anterior part is spared. Handle of malleus was, hence, preserved in PT squamous disease with cholesteatoma in our study, in as many as 77.78% of the cases, as it mainly involves sinus tympani with necrosis of incus and stapes. In cases of PF active squamous disease with cholesteatoma, almost all patients had absent head of malleus. Therefore, the ossicular involvement depends upon the origin and spread of cholesteatoma.

*Pattern of necrosis of incus in different types of COM*

In patients of PT active squamous disease with granulation, 29.41% had absent incus and 35.29% had intact incus. In patients with PF active squamous disease with cholesteatoma, incus was found to be necrosed in 92.85% cases, whereas in patients with PT active squamous disease with cholesteatoma it was necrosed in 88.89% cases. In PF cholesteatoma cases, incus was absent in 64.29% patients and in PT cholesteatoma cases, it was absent in all 88.89% cases. Hence incus necrosis is more common in PT cholesteatoma than in PF cholesteatoma, due to the posterior spread of cholesteatoma. It is also more common in cases with cholesteatoma than with granulation disease.

*Pattern of necrosis of stapes suprastructure in different types of COM*

In 64.7% patients with PT active squamous disease with granulation, stapes was found to be intact. One interesting finding in our study is that PT active squamous disease with cholesteatoma and PF active squamous disease with cholesteatoma had a similar incidence of stapes necrosis, unlike incus necrosis. Stapes suprastructure was found to be necrosed in 88.89% patients with PT cholesteatoma, and in PF cholesteatoma it was found to be necrosed in 85.72% cases. If the necrosis depends only upon the pattern of spread, the incidence of stapes necrosis should be higher in PT cholesteatoma than PF. Therefore, it appears that the stapes suprastructure is more vulnerable to necrosis by cholesteatoma in comparison to the incus. Further studies need to be undertaken in this regard.

## Conclusion

The degree of ossicular necrosis has a positive correlation with the type of disease pathology, being higher in squamous disease than in mucosal disease. It is highest in squamous disease with cholesteatoma, followed by squamous disease with granulations, mucosal disease with granulations, and inactive mucosal disease, in that order. The pattern of ossicular necrosis varies with the site of origin of the disease and the pattern of spread of cholesteatoma. Stapes appears to be more vulnerable to destruction by cholesteatoma as compared with incus, whereas incus was found to be the most vulnerable ossicle for erosion overall. Active squamous COM of PT may be associated with exclusive granulation disease, without cholesteatoma, and lead to ossicular necrosis.
